# Treatment of Cystitis by Hungarian General Practitioners: A Prospective Observational Study

**DOI:** 10.3389/fphar.2019.01498

**Published:** 2019-12-19

**Authors:** Ria Benko, Maria Matuz, Zoltan Juhasz, Julia Bognar, Reka Bordas, Gyongyver Soos, Edit Hajdu, Zoltan Peto

**Affiliations:** ^1^ Department of Clinical Pharmacy, University of Szeged, Szeged, Hungary; ^2^ First Department of Internal Medicine, Infectology Unit, University of Szeged, Szeged, Hungary; ^3^ Grove Lodge One, Norwich, United Kingdom; ^4^ Emergency Department, University of Szeged, Szeged, Hungary

**Keywords:** lower urinary tract infection, management, antibiotic choice, fluoroquinolone use, symptomatic treatment, antibiotic stewardship

## Abstract

**Background:** Lower urinary tract infections (LUTIs) are amongst the most common community acquired infections with frequent antibiotic prescribing.

**Objectives:** To assess empiric antibiotic choice in different types of lower urinary tract infections. We also aimed to identify determinants of fluoroquinolone prescribing, as well as to determine the rate of short antibiotic courses. The frequencies of executing laboratory tests and recommending analgesics/anti-inflammatory drugs were also assessed.

**Methods:** A prospective observational study was performed in 19 different Hungarian primary care practices. Participating general practitioners (GPs) filled out data sheets for each patient with a suspected urinary tract infection. Details of drug use were evaluated. Comparison of different LUTI groups were made by descriptive statistics and univariate analysis. Possible determinants of fluoroquinolone prescribing were assessed by logistic regression.

**Results:** Data sheets of 372 patients were analyzed. The majority of patients (68.82%) had acute uncomplicated cystitis. While antibiotics were prescribed for almost every patient (uncomplicated cases: 92.58%, complicated cases: 94.83%), analgesics/anti-inflammatory drugs were recommended at a rate of 7.81% in uncomplicated, and 13.79% in complicated cystitis cases. Ciprofloxacin was the most commonly prescribed antibacterial agent in both types of cystitis. Short-term antibiotic therapy was prescribed in one third of relevant cases. Logistic regression found a weak association between fluoroquinolone use and patient’s age and presence of complicating factors.

**Conclusions:** Many aspects of suboptimal cystitis management were identified (e.g. unnecessarily broad spectra agents, too long antibiotic courses). In this study, patient characteristics has weakly influenced fluoroquinolone prescribing. Based on these results there is considerable room for improvement in primary care antibiotic therapy of cystitis in Hungary.

## Introduction

To combat antibiotic resistance by lowering selection pressure, rationalizing antibiotic use for the most frequent community infections should be the key target of any antibiotic stewardship programmes. Lower urinary tract infections (LUTIs) are among the most commonly encountered illnesses in the ambulatory care (e.g. almost half of all women will experience at least one episode of cystitis during their lifetime), with consequently high antibiotic prescribing (Bonkat et al., 2017; Tyrstrup et al., 2017; Dumpis et al., 2018).

The classification of urinary tract infections is based on the affected anatomical site, and can be further stratified into uncomplicated or complicated types based on the presence or absence of complicating factors (e.g. diabetes mellitus) (Bonkat et al., 2017; Reynard and Biers, 2019). Compared to uncomplicated LUTI, the pathogen spectrum of complicated LUTIs is much wider, and these bacteria are more likely to be resistant to antibiotics, thus their empirical treatment tends to utilize broad-spectrum antibiotics (Bonkat et al., 2017). Unfortunately, the widely used coding system of diseases (International Statistical Classification of Diseases and Related Health Problems (ICD) version 10) does not follow this classification, which limits the capabilities of database studies. Previous drug utilisation studies based on the national health care database of Hungary revealed suboptimal antibiotic use in various infections (Juhasz et al., 2013; Matuz et al., 2015). The aim of this study was to overcome the limitations of database studies by exploiting clinical data that enables correct diagnosing and further stratification of cases. Based on these exact patient-specific data we have assessed the empiric antibiotic choice in complicated and uncomplicated LUTIs. Determinants of fluoroquinolone prescribing, the use of analgesics/anti-inflammatory drugs and the frequency of laboratory urine analysis (microbiological and chemical) were also evaluated.

## Methods

A prospective observational study was conducted. General practitioners (GPs) were invited and voluntarily participated, without any financial incentives. Twenty-five GPs accepted participation (response rate: 51%), and 19 GPs (i.e. 19 different primary care unit from various geographical location) contributed throughout the study. GPs were asked to fill in a data sheet for all consecutive patients who first presented with a suspected urinary tract infection. The following data were recorded: patient’s demography, drug allergy, presence of complicating factors, clinical signs, history of urinary tract infections in the previous year, performed lab tests and treatments. Doctors’ specialty and experience (number of practicing years) were derived from a public national database (National Health Registration and Training Centre).

All patients over 16 years of age were eligible for inclusion. Pregnant women, as well as cases with a suspected upper UTI (pyelonephritis), or genital or sexually transmitted infection (e.g. vaginal discharge) and cases where crucial data (e.g. prescribed/recommended therapy) were missing were excluded. Based on the recorded signs/complaints and comorbidities we classified each case as an uncomplicated or complicated lower urinary tract infection according to the guideline of the European Urological Association (Bonkat et al., 2017). The followings were considered as complicating factors: male gender, diabetes mellitus, presence of indwelling catheter/stent/tube, reconstruction of the urinary tract, recent instrumentational intervention within the urinary tract, functional/anatomical defects (e.g. obstruction, incontinence), renal failure, kidney transplant, and immunosuppression. In accordance with the widely accepted definition, we defined recurrent cystitis as a minimum of 2 infectious episodes within 6 months, or 3 infectious episodes within a year (Bonkat et al., 2017). Antibiotics were classified according to the WHO ATC (Anatomical-Therapeutic-Chemical) index (version 2019). Short-term antibiotic course was defined as prescribing a single dose of fosfomycin, a 3-day-course of fluoroquinolone, a 5-day-course of beta-lactam and 5–7 days of nitrofurantoin therapy for uncomplicated cystitis (Reynard and Biers, 2019).

Patient age, diagnostic measures and prescribed/recommended therapy was compared in complicated and uncomplicated cystitis by descriptive methods and univariate analysis (Fischer’s and Welch’s tests). Potential influencing factors of fluoroquinolone prescribing (patient-specific characteristics: age, gender, recurrent cystitis, presence of complicating factors; GP-specific factors: years of practicing, and specializations) were analyzed by logistic regression. Statistical analyses were performed with the R statistical software (version 3.5).

The study protocol was approved by the Regional Human Medical Biology Research Ethical Board of the University of Szeged, Hungary. The ethical approval did not allowed the identification and follow-up of patients, hence the therapeutic outcomes could not be evaluated.

## Results

Overall, 510 data sheets were collected. A total of 138 patients were excluded (because of pregnancy, n = 11; genital/sexually transmitted infection, n = 11; suspected kidney infection, n = 74; missing data, n = 42), thus data sheets of 372 patients were analyzed. Most patients were female (n = 342, 91.94%), diagnosed with uncomplicated cystitis (n = 256, 68.82%). Recurrent cystitis was present in one fifth of patients (n = 71, 19.07%).

The average age of those diagnosed with complicated cystitis was significantly higher (64.44 ± 16.30 years vs. 48.12 ± 19.56), and recurrent cystitis was also slightly more frequent in this patient group. (**Table 1**).

**Table 1:** General patient characteristics, diagnostics and therapeutics.

		Uncomplicated cystitis N = 256 patients (100.00%)	Complicated cystitis N = 116 patients (100.00%)	P value
Gender	Male	–	30 (25.86%)	
	Female	256 (100.00%)	86 (74.14%)	–
Age (years)	Average ± SD	48.12 ± 19.56	64.44 ± 16.30	P < 0.001^1^
	>65 years (%)	57 (22.27%)	62 (53.45%)	P < 0.001^2^
Diabetes mellitus		–	37 (31.90%)	–
Functional/anatomical abnormality of the urinary tract		–	34 (29.31%)	–
Antibiotic prescribing		237 (92.58%)	110 (94.83%)	P = 0.5076^2^
Recurrent infection		33 (12.89%)	38 (32.76%)	P < 0.001^2^
Dipstick test executed		216 (84.38%)	100 (86.21%)	P = 0.755^2^
Microbiological urine analysis executed		39 (15.23%)	25 (21.55%)	P = 0.141^2^
Analgesic use		20 (7.81%)	16 (13.79%)	P = 0.0878^2^
Short-term antibiotic use*		76 (32.07%)	32 (29.09%)	P = 0.619^2^
Fluoroquinolone prescribing*		111 (46.84%)	54 (49.09%)	P = 0.729^2^

^1^Welch’s two-sample t-test. ^2^Fisher’s exact test. *Considering only those patients who were prescribed antibiotics (Uncomplicated cystitis: 237; Complicated cystitis: 110).

**Figure 1:**
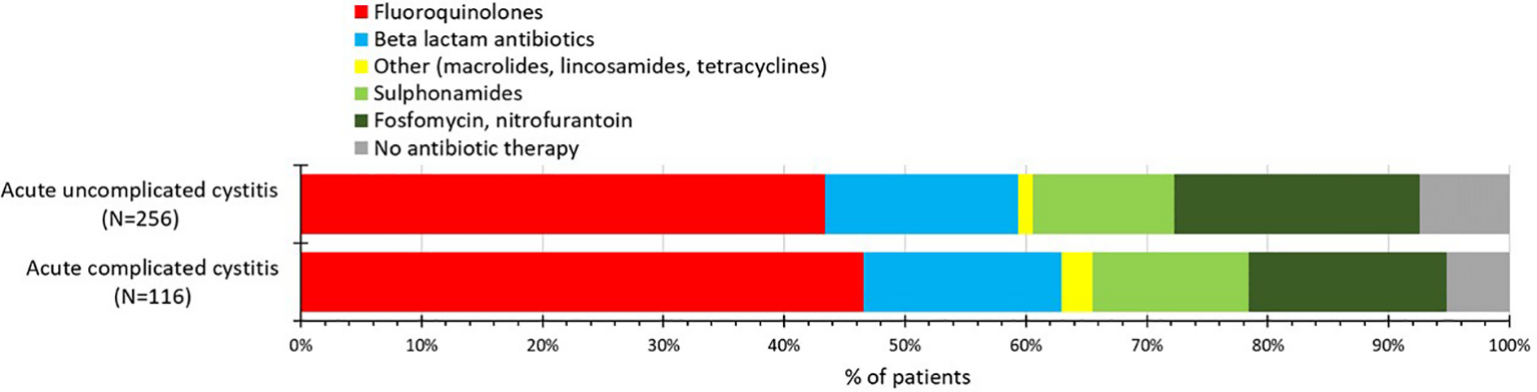
Antibiotic prescription at initial presentation.

Diagnostic measures and details of prescribed/recommended therapies are summarized in **Table 1** and **Figure 1**. Those with complicated cystitis had higher age and higher rate of recurrent infection (**Table 1**). With some exceptions, urine analysis was extensively performed (in over 85% of cases) in both cystitis types, while microbiological analysis of midstream urine was performed only in a minority of cases (15.23% in uncomplicated and 21.55% in complicated cystitis, see **Table 1**). Antibiotic was prescribed for 347 patients (93.28%), however, antibiotic prescription rate of individual GPs ranged between 50.00% and 100.00%. Analgesics/anti-inflammatory drugs was recommended in 1 per 10 cases, while individual GP’s recommendation rate ranged between 4.17% and 100.00%. Using non-antibiotic treatment only (analgesic/anti-inflammatory agent OR cranberry product OR herbal tea) was recommended in as few as 11 cases (2.96%).

The most frequently prescribed antibiotics are shown in **Table 2**. Although their rankings differed, the top five antibiotics were the same in both types of cystitis. Ciprofloxacin was the most widely used agent. Sulphamethoxazol-trimethoprim and fosfomycin were among the most frequently prescribed antibacterials. Nitrofurantoin was prescribed rarely (uncomplicated cystitis: 4 cases, complicated cystitis: 6 cases). Guideline-recommended short-term antibiotic course was initiated in ~30% of cases (**Table 2**).

**Table 2:** Most frequently prescribed systemic antibiotics.

	Uncomplicated cystitis (N = 256 patient; 100.00%)			Complicated cystitis (N = 116 patient; 100.00%)		
	Active agent	patient	%	Active agent	patient	%
1	Ciprofloxacin	55	21.48	Ciprofloxacin	30	25.86
2	Fosfomycin	48	18.75	Norfloxacin	22	18.97
3	Norfloxacin	48	18.75	SMX-TMP	15	12.93
4	SMX-TMP	30	11.72	Fosfomycin	13	11.21
	AMC	14	5.47	Cefuroxime	9	7.76
5	Cefuroxime	14	5.47			

AMC, amoxicillin-clavulanic acid. SMX-TMP, sulphamethoxazol-trimethoprim.

Only evidence of a weak association was found between patient characteristics and fluoroquinolone prescribing (**Table 3**): younger adults and those with complicating factors tended to be treated with fluoroquinolones (odds ratio *age*: 0.98 yearly; odds ratio *complicating factors*: 1.80). The frequency of fluoroquinolone prescribing varied substantially: some of the GPs did not prescribe these agents at all, while a single GP prescribed this antibiotic in 85.71% of cystitis cases diagnosed in her practice.

**Table 3:** Fluoroquinolone prescribing with regard to patient- and GP-specific factors.

		No fluoroquinolone prescribed N = 182 patients (100.00%)	Fluoroquinolone therapy prescribed N = 165 patients (100.00%)	Logistic regression	
				OR (95% CI)	P value		
Gender	Female	170 (93.41%)	150 (90.91%)	baseline	
	Male	12 (6.59%)	15 (9.09%)	1.52 (0.67–3.46)	0.319
Age (years)	Average ± SD	56.40 ± 19.51	50.95 ± 20.38	0.98 (0.97–0.99)	0.002
Complicating factors		49 (26.92%)	50 (30.30%)	1.80 (1.03–3.12)	0.038
Recurrent LUTI		39 (21.43%)	28 (16.97%)	0.65 (0.36–1.17)	0.153
GP’s experience	10–20 years of practice	40 (21.98%)	30 (18.18%)	Baseline	
	>20 years of practice	142 (78.02%)	135 (81.82%)	1.35 (0.76–2.37)	0.309
GP’s specialty	Only GP specialty	85 (46.70%)	73 (44.24%)	Baseline	
	GP+other specialty	97 (53.30%)	92 (55.76%)	1.10 (0.69–1.75)	0.684

OR, Odds ratio; 95% CI, confidence interval; logistic regression model constant: 0.52; p = 0.177.

## Discussion

The burden of urinary tract infections is high worldwide (Foxman, 2014). However, field studies to provide more insight into the clinical practice of the outpatient management of UTIs in adults are scarce (Martinez et al., 2007; Llor et al., 2011; Denes et al., 2012; Butler et al., 2017; Dumpis et al., 2018). The main objective of our study was to fill in this gap by assessing empiric antibiotic choice in different lower urinary tract infections (i.e. complicated and uncomplicated cystitis). No significant difference has been observed in the antibiotic prescribing patterns for the two types of cystitis.

As antibiotics are unambiguously superior to placebo for urinary tract infections (Falagas et al., 2009), antibiotic use can be considered optimal in every LUTI case. The rate of antibiotic prescribing for LUTI in this study (over 90%) meets international quality indicators (Adriaenssens et al., 2011; Le Marechal et al., 2018) and correlates well with findings of antibiotic utilisation studies for other countries, except the Netherlands and Latvia where antibiotic prescription rate is reported to be less than 60% and around 70% in acute urinary tract infections in adults, respectively (Martinez et al., 2007; Llor et al., 2011; Butler et al., 2017). The lower prescription rate in these countries may be explained by the more frequent application of delayed or conditional antibiotic prescribing (Gagyor et al., 2012; Gagyor et al., 2015).

The use of analgesics/anti-inflammatory agents was found to be limited, which can be explained by the lack of such recommendations in national and international guidelines on urological infections (Ministry of Health, 2010; Bonkat et al., 2017). This is in contrast to the German guideline which only recommends symptomatic treatment in uncomplicated cystitis with mild to moderate symptoms (Kranz et al., 2017).

Short-course oral antibacterial treatment has been proven to be as effective as long courses of antibiotics in the ambulatory management of LUTIs (Dawson-Hahn et al., 2017). However, in our study only 1 in 3 patients with uncomplicated cystitis received a short-term antibiotic course, a finding similar to other studies (Llor et al., 2011; Denes et al., 2012; Hawker et al., 2014; Durkin et al., 2018; Phamnguyen et al., 2019). This may be explained by the fact that pharmacies can dispense only complete boxes of medicines instead of a certain number of tablets actually needed,

The GPs in this study performed urine analysis (i.e. a urine dipstick test) in the majority of cases in both types of cystitis. As dipstick analysis is considered to have little added value when typical urinary tract symptoms are present (Bonkat et al., 2017), unnecessary testing can be presumed in some cases. On the other hand, as dipstick analysis may also give information on the causative bacterial class (i.e nitrite test is positive if *Enterobacteriaceae* is present) its use can be justified.

Microbiological confirmation of UTI is recommended in all cases of complicated infections and recurrent urinary tract symptoms (Bonkat et al., 2017). Unfortunately, microbiological analysis was requested at a suboptimal rate (i.e only in 21.55% in complicated LUTI) which can be partly explained by logistic issues (lack of local laboratories and long distance between the GP’s practice and laboratories) that hamper timely identification of the pathogens (Hajdu et al., 2009). The Flexicult system (Bates et al., 2014; Hullegie et al., 2017), aimed for point-of-care diagnoses of UTI and susceptibility testing of urinary pathogens is not used in Hungary due to the lack of reimbursement (and also this is the case for other point-of-care tests utilized in other e.g. Scandinavian countries).

The pattern of antibiotic use was similar for both uncomplicated and complicated cystitis, and showed a high dominance of fluoroquinolones. The use of fluoroquinolones in UTI varies greatly in the literature, but none of the European countries (except for a non-recent publication from France)(Denes et al., 2012) reported such a high ratio of fluoroquinolone prescribing. In Norway, Denmark, Sweden and the Netherlands the use of fluoroquinolone agents were reported to be below 10%, thanks to strict and well-functioning antibiotic stewardships (Agdestein et al., 2011; Tyrstrup et al., 2017; Dumpis et al., 2018; Holm et al., 2019). In Latvia and Lithuania, fluoroquinolones are reported to be used in 23% and 25% of uncomplicated UTI cases, respectively (Dumpis et al., 2018). Similar rates for fluoroquinolone use in cystitis (22%) was reported for Belgium (Tyrstrup et al., 2017). In contrast, a relatively frequent fluoroquinolone use (30% of all cases) in uncomplicated UTI has been reported from the US recently, and this rate was even higher in the preceding years (Durkin et al., 2018; Cowart et al., 2019). The decreasing trend is explained by the publication of an FDA black box warning on fluoroquinolones in 2016 (Cowart et al., 2019). In Hungary no up-to-date guidelines are available for the antibiotic prescribing in UTIs, and the previous guidelines (that still pop-up as the first hit in a google search) recommended the use of fluoroquinolones in all types of UTIs (Nephrology, 2006; Ministry of Health, 2010). Thus, in fact, our findings are not surprising, but the high fluoroquinolone dominance is unwanted, especially if we consider the 2018 safety review-based restrictions (European Medicines Agency, 2019) of the European Commission on fluoroquinolone prescribing (valid since 11 March 2019 in all EU countries).

Only weak determinants of fluoroquinolone prescribing were identified: younger age and presence of complicating factors were found to influence the rate of fluoroquinolone therapy. As fluoroquinolones have a high potential to generate resistance (Schito et al., 2009), and the fluoroquinolone resistance of *E.coli* already exceeds 20% in urine samples in Hungary (Hungarian National Bacteriological Surveillance Management Team, National Centre for Epidemiology), the high use of fluoroquinolones demonstrated in our study is clearly worrisome. Qualitative studies are needed to better explore the high differences in fluoroquinolone prescribing rate of individual GPs.

Patients with uncomplicated cystitis were prescribed fosfomycin in 18.75% of all cases and nitrofurantoin in only 4 cases which is suboptimal according to international quality indicators (Adriaenssens et al., 2011). These data are also regarded as worrisome, as these two drugs have their renaissance in the treatment of uncomplicated LUTI due to their preserved effectiveness (Gardiner et al., 2019). On the other hand, these agents (i.e. fosfomycin and nitrofurantoin) should be avoided in complicated cystitis due to their lack of activity against the potential uropathogens in these cases (Bonkat et al., 2017). Nevertheless, fosfomycin was among the top five agents prescribed for complicated cystitis.

Regarding sulphamethoxazol-trimethoprim (SMX-TMP), its first line use should be limited to uncomplicated cases, and only when local resistance patterns permit this choice. In Hungary, the prevalence of *E. coli* strains resistant to sulphamethoxazol-trimethoprim is reported to be above 20% (Hungarian National Bacteriological Surveillance Management Team National Centre for Epidemiology), but considering that resistance surveillance systems may overestimate resistance patterns (Schmiemann et al., 2012), SMX-TMP use can be accepted as a rational choice in uncomplicated cystitis.

The strengths of our survey include the ability to exploit clinical data. Also, by applying common diagnostic and classification criteria, misclassification bias could be avoided, and the choice of antibiotic could be justified. A limitation of our study is the voluntary participation of GPs. As volunteering GPs may be more concerned about their rational antibiotic prescribing practices, the global prescribing patterns in Hungary might be more suboptimal than presented in this study.

## Conclusions

Our study has found similar patterns of antibiotic use in both types of cystitis (with high fluoroquinolone dominance), and identified suboptimal antibiotic use from various aspects. Patient characteristics has weakly influenced fluoroquinolone prescribing More prudent use of antibiotics in lower urinary tract infections is urgently needed.

## Data Availability Statement

The datasets generated for this study are available on request to the corresponding author.

## Ethics Statement

The studies involving human participants were reviewed and approved by the Regional Human Medical Biology Research Ethical Board of the University of Szeged. The patients/participants provided their written informed consent to participate in this study.

## Author Contributions

RBe, MM, ZJ, JB, EH, and ZP had the original idea for the manuscript. ZJ and EH organized data collection. RBo, MM, JB, GS, and ZP contributed to the analysis. RBo, MM, and JB drafted the manuscript, which was reviewed and approved by ZP, ZJ, GS, and EH.

## Funding

The study was funded by the University of Szeged.

## Conflict of Interest

Author JB is employed at Grove Lodge One, though she was not at the time the study was conducted.

The remaining authors declare that the research was conducted in the absence of any commercial or financial relationships that could be construed as a potential conflict of interest.
